# Novel Ecdysteroids from *Serratula wolffii*

**DOI:** 10.1100/2012/651275

**Published:** 2012-05-02

**Authors:** Attila Ványolós, Zoltán Béni, Miklós Dékány, András Simon, Mária Báthori

**Affiliations:** ^1^Department of Pharmacognosy, University of Szeged, Eötvös utca 6, 6720 Szeged, Hungary; ^2^Spectroscopic Research, Gedeon Richter Plc., Gyömrői út 19–21, 1103 Budapest, Hungary; ^3^Department of Inorganic and Analytical Chemistry, Budapest University of Technology and Economics, Szent. Gellért tér 4, 1111 Budapest, Hungary

## Abstract

Two new and one known ecdysteroids were identified in the methanolic extract of the roots of *Serratula wolffii*. The new compounds isolated were ponasterone A-22-apioside (**1**) and 3-epi-shidasterone (**3**), together with the known 3-epi-22-deoxy-20-hydroxyecdysone (**2**). The structures of compounds **1**–**3** were determined by extensive spectroscopic techniques, including one- and two-dimensional NMR methods.

## 1. Introduction

Ecdysteroids comprise a class of 5ß-androstane steroid hormones. They are widespread in invertebrate species (mainly arthropods), acting as moulting hormones responsible for metamorphosis and a variety of other processes in arthropods. They are also present in large amounts and widely distributed in many plant species (from the Asteraceae, Lamiaceae, Caryophyllaceae, and Polypodiaceae families), where they display a certain potential in defense of plants against insects and nematodes [1]. Functional analogues of ecdysteroids have been used as selective pest control agents [2].

Their ready availability in plants has led to a number of pharmacological studies which have demonstrated that they influence many physiological functions and are not toxic to mammals [3]. Probably the most promising and most extensively examined of their pharmacological activities is their anabolic action. Their documented muscle size- and strength-increasing effects display similarities with the mode of action of the androgenic steroids, but the ecdysteroids are not able to bind to mammalian nuclear receptors; accordingly, they do not exhibit androgenic, estrogenic or glucocorticoid side effects. They may be a promising alternative to anabolic steroids use in medical therapy. However, the mechanisms of action of ecdysteroids are mainly unknown; their effects on mammalian systems are possibly mediated via the vitamin D nongenomic signaling pathways [4, 5].

Because of their anabolic activity, ecdysteroids and the ecdysteroid-containing preparations are widely advertised on the Internet as growth promoters.

They attract scientific interest not only because of their prospective use in conventional therapy, but also because they have a tremendous potential in up-to-date therapy (gene-switch systems) [6].

*Serratula wolffii,* member of the *Serratula* (plumeless saw-wort) genus, biosynthesizes a wide variety of structurally different ecdysteroids and is a rich and unique source of ecdysteroids, including compounds with an extra double bond at position 20(22) (1-hydroxy-20,21-didehydrotaxisterone and 20,22-didehydrotaxisterone), with a 14*α*,15*α*-epoxide ring (14*α*,15*α*-epoxy-14,15-dihydrostachysterone B), with a furan ring in the side chain (serfurosterone A and B), or with an intramolecular ether in the side chain (shidasterone derivatives) [7–10].

As a continuation of our interest in unusual steroids of *Serratula wolffii*, the present paper gives an account of the discovery of two new compounds and the isolation and structure determination of one known compound from the roots of this plant.

## 2. Experimental Part

### 2.1. General

Column chromatography (CC): *C-Gel* octadecyl silica (0.06–0.02 *μ*m; *Chemie Uetikon*, Uetikon, Switzerland). HPLC: *Jasco-PU-2080* pump and *Jasco-2070/2075* detector; *Zorbax-Sil* column (5 *μ*m, 4.6 mm × 250 mm; *DuPont*, Paris, France). Rotation planar chromatography (RPC): *Harrison 8924-Chromatotron* instrument (*Harrison Research*, Palo Alto, CA); stationary phase, silica gel *60 GF_254_* (*E. Merck*). Optical rotations: *Perkin Elmer 341* polarimeter. UV spectra: *Shimadzu UV-2101 PC* spectrophotometer; MeOH solutions; *λ*_max⁡_ (log *ε*) in nm. NMR spectra of compounds **1** and **2** were recorded in methanol-d_4_ in a Shigemi sample tube at 298 K using a Varian 800 MHz NMR spectrometer equipped with a **1**H{13C/15N} triple-resonance ^13^C enhanced salt tolerant cold probe operating at 800 MHz for ^1^H and 201 MHz for ^13^C NMR. Spectra of compound **3** were recorded in methanol-d_4_ in a Shigemi sample tube at room temperature with Bruker Avance DRX-500 spectrometer. Chemical shifts were referenced to the solvent resonances (*δ*_C_ = 49.15 and *δ*_H_ = 3.31 ppm). The pulse programs of all experiments [^1^H, ^13^C APT, gs-HMQC, gs-HSQC, gs-COSY, zTOCSY, NOESY, ROESY] were taken from the Bruker and Varian software libraries.

High-resolution MS (HR-MS) measurements were performed on a Thermo LTQ FT Ultra spectrometer (Thermo Fisher Scientific, Bremen, Germany). The ionization method was ESI and operated in positive ion mode. The ion transfer capillary temperature was set at 280°C, and the capillary voltage was 4.7 kV for each measurement. The samples were dissolved in a MeOH-H_2_O (50-50 V/V%) + 1 V/V% cc. AcOH solution. Data acquisition and analysis were accomplished with Xcalibur software version 2.0 (Thermo Fisher Scientific Inc.).

### 2.2. Plant Material

Roots of *Serratula wolffii* Andrae were collected in August 2003, from Herencsény. A voucher specimen (collection number S94) has been deposited at the Department of Pharmacognosy, University of Szeged, Hungary.

### 2.3. Extraction and Isolation

 The fresh roots of *S. wolffii* (4,7 kg) were extracted with methanol at room temperature, and the extract was purified by precipitation using acetone. The supernatant was evaporated to dryness. The dry residue (137.5 g) was dissolved in methanol and was applied to a polyamide column (MN-polyamide SC6, Woelm, Eschwege, Germany).

The fraction (24.4 g) eluted with water from the polyamide column was subjected to low-pressure reversed-phase CC on octadecyl silica. The fraction (0.39 g) eluted with MeOH-H_2_O (50 : 50, v/v) was further purified by RPC. The fractions eluted with solvent system CH_2_Cl_2_-MeOH-C_6_H_6_ 25 : 3 : 2 were fractionated again by RPC (AcOEt-EtOH-H_2_O 20 : 2 : 1). These fractions were finally purified by normal-phase HPLC (CH_2_Cl_2_-i-PrOH-H_2_O 125 : 40 : 4, 2.5 mL/min; detection at 245 nm) to obtain compounds **1 **and **2**.

Another fraction (0.48 g) eluted from the reversed-phase column with methanol was purified with repeated RPC (CH_2_Cl_2_-MeOH-C_6_H_6_ 50 : 3 : 2 and AcOEt-EtOH-H_2_O 80 : 5 : 2). The RPC was followed by normal-phase HPLC (CH_2_Cl_2_-i-PrOH-H_2_O 125 : 40 : 3, 2.5 mL/min; detection at 245 nm) which gave compound **3**.

*PonasteroneA-22-apioside *(*2*β*,3*β*,14*α*,20R-tetrahydroxy-22- *{[*3,4-dihydroxy-4- *(*hydroxymethyl*)*-tetrahydrofuran-2-yl *]*oxy*}*-5*β*-cholest-7-en-6-one*)*; **1***: [*α*]_*D*_^27^ = +9° (*c* = 0.1, MeOH); UV (MeOH): 242 (2.81). ^1^H and ^13^C NMR (MeOH-*d*_4_): Table 1. ESI-MS: 597 (0.5, [M+H]^+^), 465 (100, [M+H-C_5_H_8_O_4_]^+^), 447 (28, [M+H-C_5_H_8_O_4_-H_2_O]^+^), 429 (15, [M+H-C_5_H_8_O_4_-H_2_O-H_2_O]^+^), 299 (5, [C_19_H_23_O_3_]). HR-ESI-MS: 597.36234 (C_32_H_53_O_10_; calc. 597.36332).

*3-Epi-22-deoxy-20-hydroxyecdysone *(*2*β*,3*α*,14*α*,20R,25-pentahydroxycholest-7-en-6-one*); ***2***: [*α*]_*D*_^27^ = +12° (*c* = 0.1, MeOH); UV (MeOH): 242 (2.42). ^1^H and ^13^C NMR (MeOH-*d*_4_): Table 1. ESI-MS: 487 (0.5, [M+Na]^+^), 469 (100, [M+Na-H2O]^+^). HR-ESI-MS: 487.30393 (C_27_H_44_O_6_Na; calc. 487.30301).

*3-Epi-shidasterone *(*22,25-epoxy-2*β*,3*α*,14*α*,20R*)*-tetrahydroxy-5*β*-cholest-7-en-6-one*)*;* ***3***: UV (MeOH): 245 (3.84). ^1^H and ^13^C NMR (MeOH-*d*_4_): Table 1. ESI-MS: 485 (15, [M+Na]^+^), 463 (41, [M+H]^+^), 445 (68, [M+H-H_2_O]^+^), 427 (13, [M+H-H_2_O-H_2_O]^+^), 345 (5) 99 [100, C_22_-C_27_]^+^). HR-ESI-MS: 462.29702 (C_27_H_42_O_6_; calc. 462.29759).

## 3. Results and Discussion

A sophisticated isolation procedure was used for the preparative-scale separation of the ecdysteroids, involving a simple clean-up through precipitation followed by multi step chromatographic methods such as column-chromatography on polyamide and octadecyl silica. The isolation was improved by the use of rotation planar chromatography and preparative HPLC.

**Figure 1:**
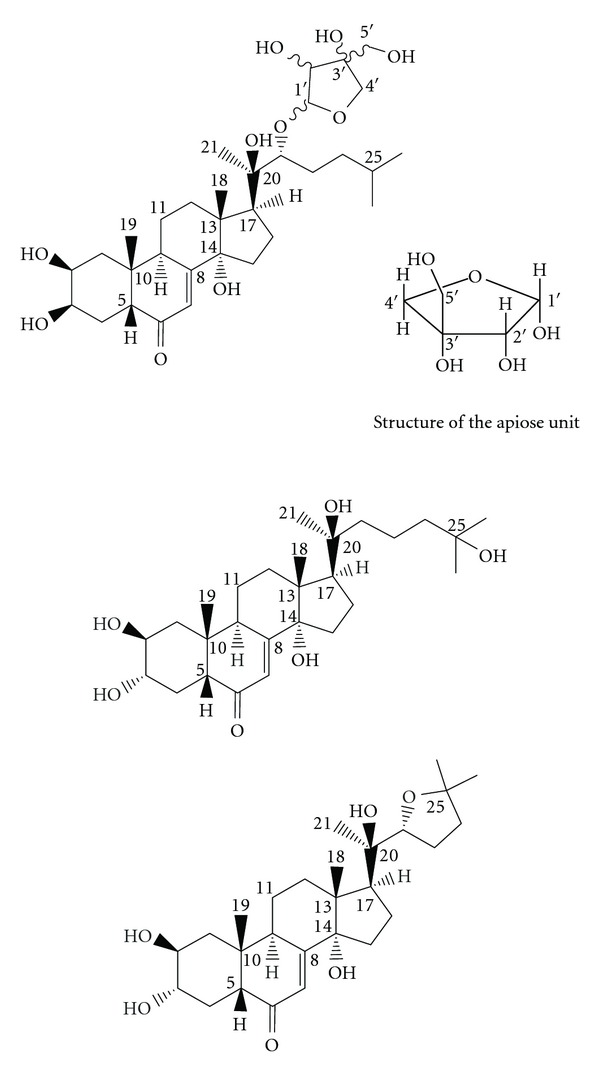
Proposed structures and numbering scheme for **1 **(top), **2** (middle) and **3 **(bottom).

The ^1^H and ^13^C NMR data on compounds **1**–**3** are given in Table 1. Our long-time intense research interest in the structure elucidation of ecdysteroids has allowed us to set up a complete database containing the characteristic ^1^H and ^13^C chemical shifts, ^1^H multiplicity and coupling constants of the compounds investigated to date. With the aid of this database and that available online (http://www.ecdybase.org/), the collected HR-MS data and one- and two-dimensional NMR data were utilized for the structural characterization of **1**–**3** following well-accepted structure elucidation strategies for related compounds. The structures of these compounds are depicted in Figure 1. 

For **1** HR-ESI-MS indicated the molecular formula C_32_H_53_O_10_ with the molecular ion peak at m/z 597.36234 ([M+H]^+^). ^1^H and ^13^C NMR assignments of the fused ring skeleton were first made from the characteristic two- and three-bond HMBC correlations of the methyl resonances, followed by analysis of the TOCSY and NOESY correlations of H-5, H-17, and H-8. The spectral data (Table 1) were in good agreement with those reported for ponasterone A-22-glycolate [11] and ponasterone A (except those relating to H-22 and C-22) [12]. Besides the skeletal resonances, the ^13^C and HSQC NMR spectra confirmed the presence of two oxymethylene groups, two oxymethine groups and an oxygen-linked tetrasubstituted carbon atom. TOCSY and HMBC correlations demonstrated that these resonances were those of an apiose unit. In agreement with the downfield-shifted C-22 signal (as compared with that observed in ponasterone A), the HMBC correlations between H-22 and C-1′ and that between H-1′ and C-22 confirmed that the apiose moiety was connected to the oxygen atom linked to C-22. The relative configuration of the sugar moiety (Figure 1) was determined from the coupling constants of 3.6 Hz between H-1′ and H-2′ and the NOE correlations between H-1′ and H-2′ and between H-2′ and H-5′. The coupling constants (10.6 and 1.6 Hz) observed between H-22 and the two H-23 protons were similar to those in ponasterone A [12] and ponasterone A-22 glycolate [11], indicating that C-22 has the same configuration in these compounds. The absolute configuration of the sugar moiety could not be determined. The proposed structure (Figure 1) was in accordance with the fragment ions detected in the HR-MS-MS spectrum, indicating loss of the sugar moiety followed by successive losses of water molecules: 465 ([M+H-C_5_H_8_O_4_]^+^), 447 ([M+H-C_5_H_8_O_4_-H_2_O]^+^), 429 ([M+H-C_5_H_8_O_4_-H_2_O-H_2_O]^+^) and 299 ([C_19_H_23_O_3_]).

The analyses of the MS and NMR data on **2** and **3** via the above protocol revealed that these two compounds have the same steroid skeleton and differ only in the side chains. The assignments of the steroid skeleton were in good agreement with the available NMR data on 3-epi-20-hydroxyecdysone [13].

For **3,** the COSY and HMQC correlations and the similarity of ^1^H and ^13^C chemical shifts in the side chain to that earlier reported for 11*α*-hydroxyshidasterone [10], in accordance with the elemental composition determined from the HR-MS data, indicated that **3** was 3-epi-shidasterone. Its molecular formula, C_27_H_42_O_6_, was established via the molecular ion peak observed by HRESI-MS/MS.

In accordance with the elemental composition C_27_H_44_O_6_ derived from HR-MS measurements (see Experimental part), the HMBC correlations of H_3_-21, H_3_-26, and H_3_-27 suggested that **2** was 3-epi-22-deoxy-20-hydroxyecdysone. This compound was earlier isolated from diapause eggs of the silkworm *Bombyx mori*, but only partial ^1^H NMR data supporting the proposed structure were reported [14]. The complete ^1^H and ^13^C NMR assignments are thus listed in Table 1.

## 4. Conclusions

Ecdysteroids containing a 3*α*-hydroxyl group are relatively rare. The number of such reported 3-epi-ecdysteroids is 21, whereas there are around 350 known ecdysteroids. The presence of these 3-epimers in plants is rather unusual; they are mainly biosynthesized in insects. In contrast with the 3-epi-ecdysteroids, the other epi-ecdysteroids, such as 22-, 14-, and 25-epi-ecdysteroids, occur only in plant species. We earlier isolated such kind of 3-epimers from *S. wolffii*: 3-epi-20-hydroxyecdysone and two structurally related 3-epi-ecdysteroids, these latter lacking the characteristic 14-hydroxy group [10].

The ecdysteroids in which the side chain forms an intramolecular ether ring constitute only a small group of compounds; they are characteristic only of plants. The well-known member of this group is shidasterone, which is fairly common in plants (*Ajuga*, *Leuzea*, *Polypodium*, *Blechnum*, *Stachyurus* and *Vitex* species). However, its derivatives are rare in plants. 11*α*-Hydroxyshidasterone and the 24-methylene-shidasterone were earlier obtained from *S. wolffii*, and we have now isolated a new shidasterone derivative (**3**) from the same species [8, 10]. Only three other shidasterone derivatives have been reported from *Polypodium vulgare* and *Vitex canescens *[15, 16].

Ponasterone-22-apioside (**1**) is the first known ecdysteroid glycoside in which apiose is attached as a sugar unit to the aglycone.

## Figures and Tables

**Table 1:** ^1^H and ^13^C NMR data of **1**, **2**, and **3**.

		**1**			**2**			**3**		
No.		^13^C	^1^H	m; *J* (Hz)	^13^C	^1^H	m; *J* (Hz)	^13^C	^1^H	m; *J* (Hz)
1	*α*	37.5	1.79	m	43.2	2.09	dd; 13.7, 4.6	43.2	2.09	dd; 13.5, 4.2
	*β*		1.43	dd; 13.1, 12.0		1.09	dd; 13.7, 11.9		1.08	dd; 13.5, 11.9
2	*α*	68.9	3.84	ddd; 12.0, 4.1, 3.4	72.3	3.64	ddd; 11.9, 9.0, 4.6	72.3	3.64	ddd; 11.9, 8.8, 4.2
3	*α*	68.7	3.95	m	75.7			75.5		
	*β*					3.34	ddd; 11.6, 9.0, 4.4		3.35	m
4	*α*	33.0	1.75	m	33.8	1.57	td; 13.1, 11.6	33.8	1.56	td; 13.0, 11.5
	*β*		1.70	m		1.75	dt; 13.1, 4.4		1.76	
5	*β*	52.0	2.38	dd; 13.0, 4.1	57.6	2.09	dd; 13.1, 4.4	57.6	2.09	dd; 13.5, 4.2
6		206.6	—		204.8	—		204.8	—	
7		122.3	5.81	d; 2.6	122.0	5.82	d; 2.8	122.1	5.82	d; 2.7
8		168.1	—		168.4	—		168.2	—	
9	*α*	35.2	3.15	ddd; 10.8, 7.4, 2.5	36.1	3.17	ddd; 11.0, 7.2, 2.4	36.1	3.17	ddd; 11.7, 7.4, 2.7
10		39.4	—		39.8	—		39.7	—	
11	*α*	21.7	1.81	m	21.6	1.81	m	21.7	1.82	m
	*β*		1.69	m		1.69	qd; 13.1, 4.9		1.70	m
12	*α*	32.8	2.11	td; 12.9, 4.7	32.5	2.14	td; 13.1, 5.0	32.5	2.16	td; 13.0, 4.5
	*β*		1.88	ddd; 12.9, 4.7, 1.8		1.86	m		1.86	m
13		48.8	—		48.3	—		48.5	—	
14		85.4	—		85.5	—		85.3	—	
15	*α*	31.9	1.58	m	31.9	1.62	ddd; 11.9, 9.6, 2.0	31.9	1.61	m
	*β*		1.98	m		1.95	m		1.97	dd; 12.7, 6.4
16		21.5	1.67	m	22.1	1.86	m	21.9	1.82	m
			2.01	m		1.90	m		2.02	dtm; 12.6, 10.0
17	*α*	51.2	2.30	t; 9.0	53.5	2.35	t; 9.0	52.0	2.37	t; 9.2
18	*β*	18.3	0.88	s	18.3	0.86	s	18.3	0.85	s
19	*β*	24.5	0.97	s	24.0	0.95	s	24.0	0.95	s
20		77.3	—		76.1	—		77.2	—	
21		22.6	1.195	s	26.7	1.28	s	20.9	1.22	s
22	a	90.2	3.35	dd; 10.6, 1.6	46.0	1.36	m	85.7	3.92	dd; 8.4, 6.2
	b					1.51	m			
23	a	30.8	1.37	m	21.9	1.42	m	28.6	1.76	m
	b		1.61	m		1.42	m		1.90	m
24	a	36.9	1.25	m	45.6	1.43	m	39.8	1.75	m
	b		1.51	m		1.43	m		1.75	m
25		29.4	1.55	qui; 6.7	71.6	—		81.9	—	
26		22.8	0.92	d; 6.7	29.3	1.19	s	28.5	1.24	s
27		23.5	0.93	d; 6.7	29.3	1.19	s	29.1	1.25	s
1′		113.0	4.98	d; 3.6						
2′		77.9	3.96	d; 3.6						
3′		80.3	—							
4′		74.7	3.79	d; 9.6						
			4.10	d; 9.6						
5′		64.8	3.55	d; 11.5						
			3.58	d; 11.5						
